# Characterization of E1 enzyme dependencies in mutant-*UBA1* human cells reveals UBA6 as a novel therapeutic target in VEXAS syndrome

**DOI:** 10.1038/s41375-025-02671-x

**Published:** 2025-06-30

**Authors:** Courtnee A. Clough, Claire Cunningham, Sophia Y. Philbrook, Kathleen M. Hueneman, Avery M. Sampson, Kwangmin Choi, Kenneth D. Greis, Daniel Starczynowski

**Affiliations:** 1 Division of Experimental Hematology and Cancer Biology, Cincinnati Children’s Hospital Medical Center, Cincinnati, OH USA; 2 Department of Cancer Biology, University of Cincinnati, Cincinnati, OH USA; 3 University of Cincinnati Cancer Center, Cincinnati, OH USA; 4 Department of Pediatrics, University of Cincinnati, Cincinnati, OH USA

**Keywords:** Haematological cancer, Cell signalling

## Abstract

VEXAS syndrome is a clonal hematopoietic disorder characterized by hyperinflammation, bone marrow failure, and high mortality. The molecular hallmark of VEXAS is somatic mutations at methionine 41 (M41) in the E1 ubiquitin enzyme, UBA1. These mutations induce a protein isoform switch, but the mechanisms underlying disease pathogenesis remain unclear. Here, we developed a human cell model of VEXAS syndrome by engineering the male monocytic THP1 cell line to express the common UBA1^M41V^ mutation. We found that mutant UBA1^M41V^ cells exhibit aberrant UBA1 isoform expression, increased vacuolization, and upregulation of the unfolded protein response, recapitulating key features of VEXAS. Moreover, proteomic analyses revealed dysregulated ubiquitination and proteotoxic stress in UBA1^M41V^ cells, with alterations in inflammatory and stress-response pathways. Functional studies demonstrated that UBA1^M41V^ cells were highly sensitive to genetic or pharmacological inhibition of E1 ubiquitin enzymes. Treatment with the E1 enzyme inhibitor TAK-243 preferentially suppressed colony formation of UBA1^M41V^ cells as compared to WT cells. Moreover, UBA1^M41V^ cells exhibited greater sensitivity to TAK-243 in competition assays and showed increased apoptosis. Interestingly, TAK-243 preferentially inhibited UBA6 activity over UBA1, suggesting that UBA6 may compensate for UBA1 dysfunction in UBA1^M41V^ cells. Targeting UBA6 using shRNA or the UBA6-specific inhibitor phytic acid further revealed an acquired dependency on UBA6 in UBA1^M41V^ cells. Phytic acid selectively impaired growth and colony formation in UBA1^M41V^ cells while sparing WT cells, highlighting a potential therapeutic vulnerability. Together, these findings establish a novel human model of VEXAS syndrome, identify key roles for UBA1 and UBA6 in disease pathogenesis, and demonstrate that UBA6 inhibition represents a promising therapeutic strategy for selectively targeting UBA1 mutant clones.

## Introduction

Dysregulation of ubiquitination results in aberrant protein accumulation which has been linked to cancer [1, 2]. E1-activating ubiquitin enzymes initiate the ubiquitination cascade, which, through E2-conjugating and E3-ligating enzymes, regulates protein expression and function to maintain proteome integrity [3, 4]. Therapeutic strategies targeting various aspects of protein homeostasis, such as 26S proteasomal inhibitors and E3 ligase inhibitors, have shown promise in a few blood cancers, however, refractory disease and long-term treatment toxicity illustrate the need for new therapies [5–9]. While dysregulation of E2 and E3 enzymes has been implicated in certain blood cancers [10–22], the extent to which aberrant E1 enzyme expression alters ubiquitination to drive pathogenesis in myeloid malignancies is not defined.

VEXAS (Vacuoles, E1-enzyme, X-linked, Autoinflammatory, Somatic) syndrome is a recently characterized acquired clonal hematopoietic disorder that frequently co-occurs with myelodysplastic syndrome (MDS) [23–25]. Its estimated prevalence is 1 in 4,000 males over the age of 50 [26]. VEXAS is defined by hyperinflammation and multisystem involvement, including chronic fevers, chondritis, and vasculitis. Patients with VEXAS often progress to bone marrow failure and are at high risk for infections, which contribute to the significant mortality associated with the disease [23, 26–28]. The molecular hallmark of VEXAS syndrome is somatic mutations in the hotspot region of methionine 41 (M41) in the E1 ubiquitin activating enzyme, UBA1 [23]. These mutations, which can have variant allele frequencies of up to 90% in hematopoietic stem and progenitor cells (HSPCs), are incompatible with lymphoid and erythroid differentiation, resulting in lymphopenias and macrocytic anemia [23, 29, 30].

UBA1, the most abundant E1 ubiquitin enzyme [31], is constitutively expressed in two isoforms: a nuclear UBA1a isoform initiated from M1 and a cytoplasmic UBA1b isoform initiated from M41. UBA1a contains a nuclear localization signal absent in UBA1b, but the specific roles of these isoforms in regulating the ubiquitinome remain poorly understood [32, 33]. In VEXAS syndrome, UBA1 M41 mutations result in an isoform switch from UBA1b to a truncated, nonfunctional isoform, UBA1c [23]. The mechanisms by which UBA1 mutations and the loss of cytoplasmic UBA1b alters the ubiquitinome and drives the pathogenesis of VEXAS remain unclear.

Treatment options for VEXAS are largely limited to managing inflammation with chronic glucocorticoid therapy, though refractory disease is common [25]. For patients with co-occurring MDS, treatment strategies such as ruxolitinib [34] and azacytidine [35] have shown some efficacy. However, these therapies are restricted to patients with MDS diagnoses, underscoring the urgent need for novel therapeutic strategies to target and eliminate mutant UBA1 HSPC clones.

To date, few cellular models of VEXAS syndrome have been developed [36–39]. While a recently described inducible human iPSC model provides insights [37], the induction of UBA1 mutations leads to a rapid loss of mutant clones. Similarly, patient-derived samples are difficult to culture, and limited cell numbers restrict comprehensive analyses. Here, we describe the development of a new human model of VEXAS syndrome using the male monocytic AML cell line THP1. We engineered THP1 cells to express the second most common and most pathogenic UBA1^M41V^ mutation, recapitulating key features of VEXAS syndrome, including aberrant vacuolization and dysregulated UBA1 protein expression. Proteomic and transcriptomic analyses of the model confirmed increased proteotoxic stress and inflammation in mutant cells. We further demonstrate that UBA1^M41V^ mutant cells exhibit increased sensitivity to E1 enzyme inhibition, both through shRNA-mediated knockdown and small-molecule approaches. Notably, we identified UBA6 inhibition as a unique vulnerability in UBA1 mutant cells, offering a potential therapeutic strategy for VEXAS syndrome.

## Results

### Development of a UBA1^M41V^ monocytic cell line model

**Fig. 1:**
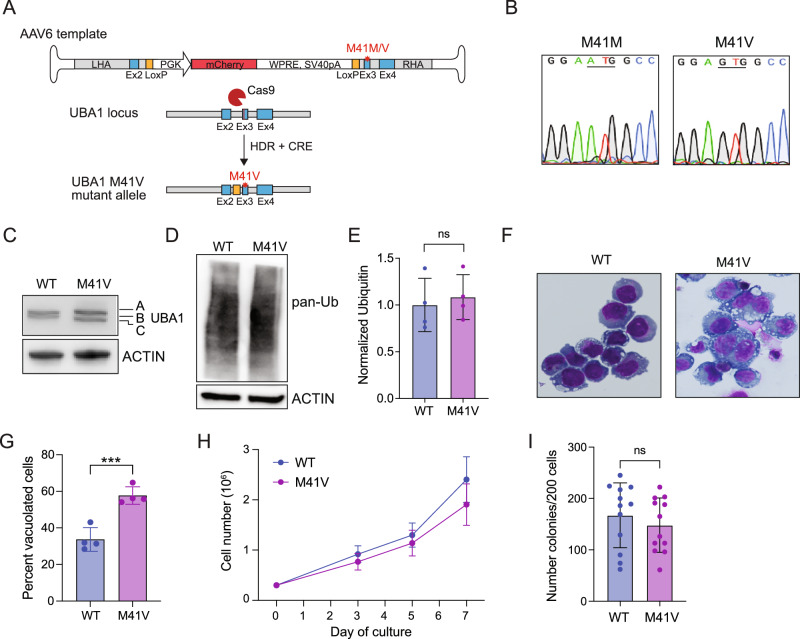
Generation of an isogenic human model of VEXAS syndrome. **A** Schematic of CRISPR/Cas9 and AAV6 mediated strategy to generate isogenic wild-type (M41M) and mutant (M41V) THP1 cell lines. Intronic regions shown in gray, exons in blue and LoxP sites in yellow. LHA left homology arm, RHA right homology arm. **B** Sanger sequencing of the M41 region of UBA1 of cDNA generated from isogenic lines. Immunoblot analysis of **C** UBA1 and **D** total ubiquitin (Ub) of WT and M41V cells. **E** Quantification of total ubiquitin from immunoblot replicates. Mean ± SD of n = 4 independent replicates. Student’s two-sided t-test was used to determine significance. **F** Representative images of vacuoles observed in WT or M41V cells stained with Wright-Giemsa. **G** Quantification of vacuolization of WT and M41V cells. Mean ± SD of n = 4 independent replicates. Student’s two-sided t-test was used to determine significance. **H** Growth curve analysis of WT and M41V cells. Mean ± SD of n = 4 independent replicates. Student’s two-sided t-test was used to determine significance. **I** Quantification of clonogenic potential of WT and M41V cells. Mean ± SD of n = 4 independent replicates (3 technical replicates per experiment). Student’s two-sided t-test was used to determine significance. *P < 0.05; **P < 0.01; ***P < 0.001.

We employed a CRISPR/Cas9 and adeno-associated virus (AAV) strategy to introduce the common VEXAS mutation, UBA1^M41V^ (M41V) (c.121 A > G), into the human monocytic AML cell line THP1. A repair template preserving the UBA1^M41M^ wild-type [40] sequence was used to generate isogenic WT control cells. Homology arms were designed to incorporate a LoxP-flanked intronic PGK.mCherry element, enabling edited cells to be tracked via flow cytometry and mCherry to be excised by transfecting CRE recombinase (Fig. 1A). We confirmed the expression of the UBA1^M41V^ mutation through sequencing of cDNA generated from the isogenic clones (Fig. 1B). Exome sequencing revealed no mutations in relevant hematopoietic genes (Supplementary Table 1). WT UBA1 is expressed as two isoforms, UBA1a and a shorter UBA1b [32, 33]. M41 substitutions result in an aberrant isoform switch from UBA1b to a nonfunctional, shorter UBA1c isoform encoded from M67 [23]. Immunoblotting confirmed that mutant cells underwent the expected UBA1b-to-UBA1c isoform switch in M41V cells (Fig. 1C). Unlike VEXAS patient samples, which exhibit a modest decrease in ubiquitination [23], M41V cells did not have global differences in total ubiquitination (Fig. 1D, E). Vacuolization, a key hallmark of mutant UBA1 cells in VEXAS patients [23, 41], was assessed in the isogenic cell lines following Wright-Giemsa staining. While WT cells had baseline vacuolization of ~30%, we observed a significant ~25% increase in vacuolization in mutant M41V cells (Fig. 1F, G, Supplementary Fig. 1D). We next evaluated whether M41V mutations affected cellular viability. Mutant cells showed a modest ~20% reduction in total cell growth compared to WT cells (Fig. 1H). Although we observed no significant difference in Annexin V+ positivity between the isogenic cell lines (Supplementary Fig. 1A), we detected a modest expansion in the sub-G1 population in M41V cells (Supplementary Fig. 1B, C). No other significant differences in cell cycle distribution were observed. Similarly, we noted a slight but insignificant ~10% decrease in the colony-forming potential of M41V cells (Fig. 1I). Taken together, mutant UBA1^M41V^ THP1 cells recapitulated key features of VEXAS syndrome, including elevated vacuolization, modestly increased apoptosis, and reduced cellular growth and colony potential. Notably, these phenotypes were observed despite the absence of a global change in total ubiquitination, suggesting that loss of ubiquitination is unlikely to be the primary driver of VEXAS pathogenesis.

### Multiomics analysis reveals proteotoxic stress and inflammatory signatures in UBA1^M41V^ cells

**Fig. 2:**
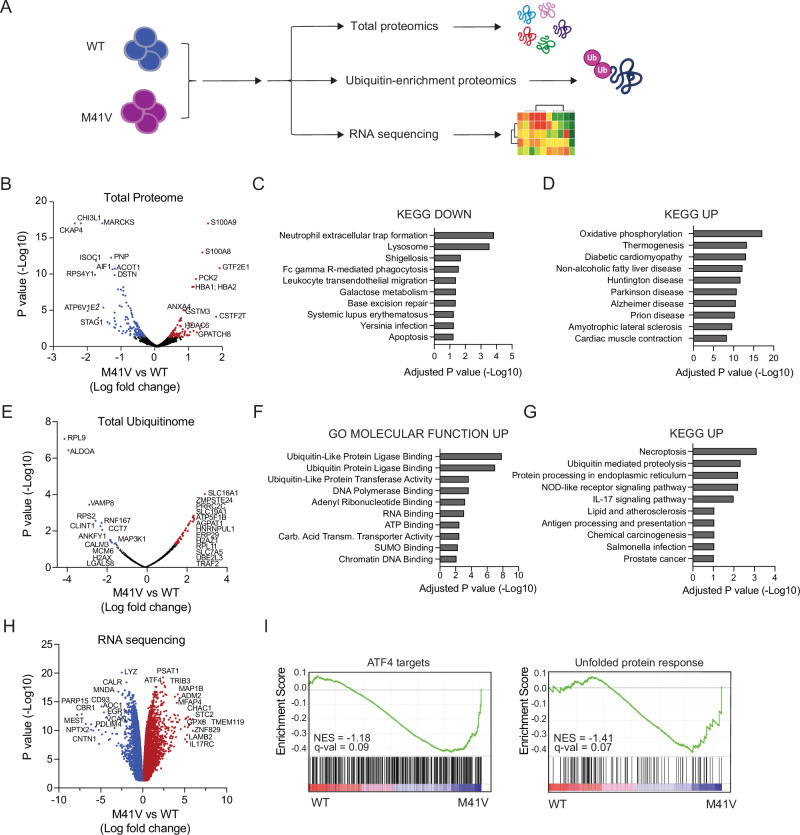
Multiomic characterization of an isogenic human model of VEXAS syndrome. **A** Overview of total proteomics, ubiquitin-enriched proteomics, and RNA sequencing that were performed on WT and M41V cells. **B** Volcano plot of differential proteomic expression of M41V cells compared to WT control (n = 3 independently collected samples). KEGG analysis of significantly downregulated (**C**) and upregulated (**D**) pathways in M41V cells compared to WT control. **E** Volcano plot of differential protein ubiquitination in M41V cells compared to WT control (n = 2 independently collected samples). **F** Upregulated GO molecular function pathways in M41V cells compared to WT control. **G** Upregulated KEGG pathways in M41V cells compared to WT control. **H** Volcano plot of differential gene expression in M41V cells compared to WT control (n = 3 independently collected samples). **I** GSEA analysis of ATF4 targets and unfolded protein response in WT and M41V cells.

We hypothesized that UBA1 mutations dysregulate UBA1 function, resulting in altered ubiquitination and changes in protein expression of specific substrates. To test this hypothesis, we expanded WT and M41V mutant cells and measured global protein expression (Fig. 2A). Total proteome analysis was refined to include 2,236 proteins detected in both WT and M41V cells (Supplementary Table 2). We identified only 158 significantly downregulated and 110 significantly upregulated proteins in M41V cells (Fig. 2B, Supplementary Table 3). KEGG (Kyoto Encyclopedia of Genes and Genomes) analysis of downregulated proteins revealed enrichment in pathways related to the inflammatory response and apoptosis (Fig. 2C). Consistent with this, we observed strong downregulation of AIF1 [42] and ISOC1 [43] proteins in M41V cells, suggesting that apoptotic and inflammatory responses were altered (Fig. 2B). KEGG analysis of significantly upregulated proteins in M41V cells indicated enrichment of pathways associated with oxidative and proteotoxic stress (Fig. 2D). Upregulated proteins, such as CAL complex proteins, S100A9, S100A8, and HDAC6 (Fig. 2B), suggested increased inflammatory pathway activation in the M41V cells, consistent with observations in VEXAS patients [44]. Additionally, the upregulation of PCK2 and EIF2B3 (Fig. 2B) in the M41V cells suggested enhanced ER and integrated stress responses, as previously reported in VEXAS patients [37, 38].

Ubiquitin modifications, primarily K48 or K63 linkages, regulate the proteome through proteasomal degradation and functional protein modulation, respectively [4, 45, 46]. To investigate this, we expanded the isogenic WT and M41V cells in the absence of proteasomal inhibitors, ensuring that most ubiquitinated peptides collected reflected K63 functional modifications, as K48 protein modifications undergo rapid degradation (Fig. 2A). Similar to our total proteome analysis, we limited this analysis to 980 peptides detected in both genotypes (Supplementary Table 4). We identified 44 ubiquitinated peptides significantly upregulated in M41V cells, including key ubiquitin regulators of NF-kB signaling and inflammation, such as the E2 ubiquitin-conjugating enzyme UBE2L3 and the E3 RING ligase TRAF2 [47, 48] (Supplementary Table 5). We also observed upregulation of the stress granule mediator PRRC2C, suggesting a potential adaptation to cellular stress induced by the UBA1^M41V^ mutation [49] (Fig. 2E). Gene ontology analysis revealed significant upregulation of ubiquitin and ubiquitin-like protein binding in M41V cells, indicating altered ubiquitin processing (Fig. 2F). Similarly, KEGG analysis showed significant enrichment in ubiquitin-mediated proteolysis and inflammatory mediators in M41V cells (Fig. 2G). In contrast, only 13 significantly downregulated ubiquitinated proteins were observed in M41V cells (Supplementary Table 5). These included MCM6, ALDOA, CALM3, and RNF167, which have been implicated in cell cycle regulation [50–53] (Fig. 2E). These alterations in cell cycle regulation may contribute to the slight differences in baseline growth observed in this model (Fig. 1H). These findings suggest that the UBA1^M41V^ mutation does not result in a global reduction in protein ubiquitination, but rather induces changes in the ubiquitination of specific proteins.

To further examine the impact of UBA1^M41V^ mutations on global gene expression, we performed RNA sequencing on M41V cells and compared them to WT controls (Fig. 2A). We identified approximately 4,600 significantly downregulated and 4,600 significantly upregulated genes in mutant cells relative to WT controls (Supplementary Table 6). Given the significant enrichment of proteotoxic diseases in our proteomic analysis (Fig. 2D), we investigated genes related to the unfolded protein response. We observed significant upregulation of ATF4 (Fig. 2H), consistent with findings in VEXAS patient samples [37, 38]. Furthermore, we noted significant upregulation of ATF4 targets [54] and unfolded protein response genes in M41V cells (Fig. 2I). Additionally, we confirmed enrichment of necroptosis [12] and ubiquitin-mediated proteolysis genes, as observed in our proteomic data (Supplementary Fig. 2B) and upregulation of tricarboxylic acid (TCA) pathways (Supplementary Fig. 2A). Paradoxically, we did not observe upregulation of immune-related genes in M41V cells as compared to WT controls (Supplementary Fig. 2A). Similarly, we did not observe increased canonical NF-κB signaling in M41V cells as compared to WT cells (Supplementary Fig. 2C). To further evaluate the expression of immune-related genes, we investigated whether any transcription factors were significantly altered in M41V cells. The proteomic analysis revealed five transcription factors that were differentially expressed compared to WT cells (Supplementary Fig. 2D). Among these, NFKB1, which encodes the p100/p50 subunit of NF-κB and typically functions as transcriptional repressor as homodimers [55], was the only transcription factor significantly upregulated. Consistent with this, we observed a corresponding decrease in canonical NF-κB target genes (Supplementary Fig. 2E). Although a hyperinflammatory phenotype was expected in M41V cells, it is possible that mutant cells downregulate or alter the expression of immune response genes to mitigate inflammatory stress [19, 56]. Recent reports suggest that, in VEXAS patients, UBA1 mutant cells suppress immune response genes, while inflammatory stress leads to the death of WT clones [39]. As these inflammatory pathways play a key role in immune cell regulation, we next evaluated the differentiation potential of WT and M41V cells. Differentiation of THP1 cells into macrophage-like cells can be induced with PMA (phorbol 12-myristate 13-acetate) [57]. WT and M41V cells were cultured with PMA or vehicle control, and the expression of CD14 and CD11b, markers of myeloid differentiation, was analyzed. At baseline, both WT and M41V cells exhibited high CD14 expression (Supplementary Fig. 2F). However, both WT and M41V cells showed minimal CD11b expression at baseline. Strikingly, while PMA strongly induced CD11b expression in WT cells, M41V cells had minimal induction (Supplementary Fig. 2F, G). These findings suggest that UBA1^M41V^-mediated downregulation of immune signaling disrupts proper immune cell function and differentiation potential.

### UBA1^M41V^ and WT cells are equally sensitive to loss of UBA1

**Fig. 3:**
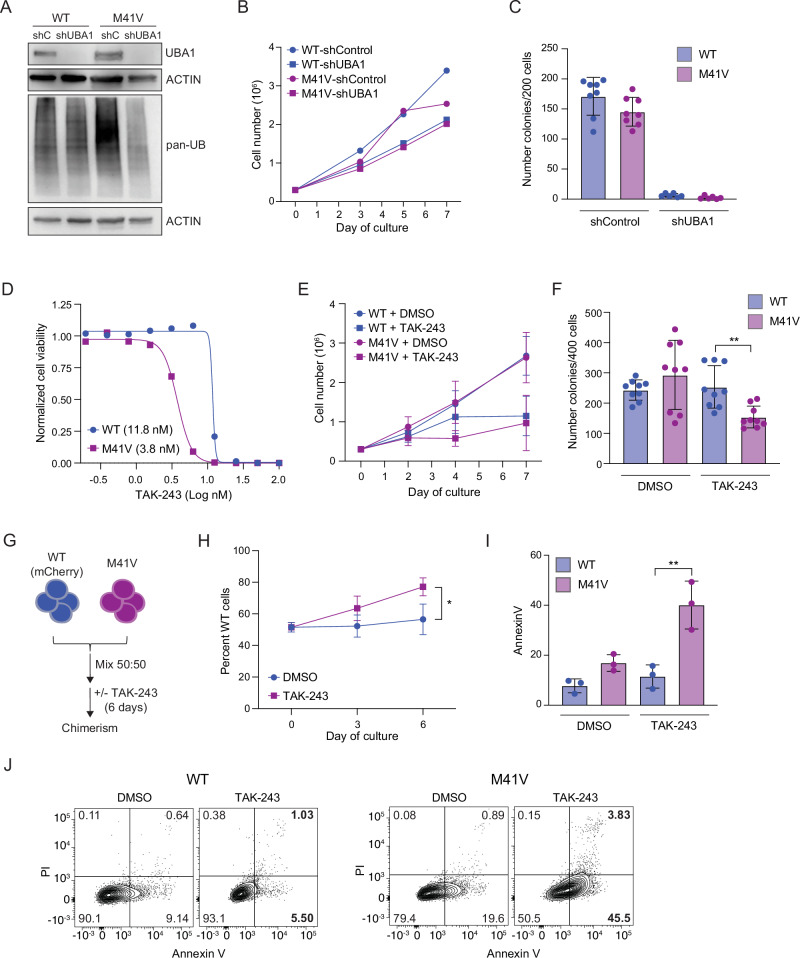
UBA1-mutant cells are preferentially sensitive to the E1 inhibitor TAK-243*.* **A** Immunoblot analysis of UBA1 and global ubiquitin in WT and M41V cells expressing a control shRNA (shControl, shC) or an shRNA targeting UBA1 (shUBA1). **B** Cell growth analysis of cells as described in (**A**). Mean of n = 6 independent experiments. **C** Quantification of clonogenic potential of WT and M41V cells expressing shControl or shUBA1. Mean ± SD, n = 8 replicates from 4 independent infections for shControl and n = 6 from 3 independent infections for shUBA1. **D** Representative cell viability analysis using CellTiter-Glo of WT and M41V cells treated with TAK-243 for 72 hours. N = 1, line of best fit is plotted. **E** Cell growth analysis of WT and M41V cells treated with vehicle control (DMSO) or TAK-243 (7.5 nM). Mean ± SD, n = 5 independent experiments. **F** Quantification of clonogenic potential of WT and M41V cells treated with vehicle control (DMSO) or TAK-243 (7.5 nM) for 48 hours prior to plating into methylcellulose containing DMSO or TAK-243 (7.5 nM). Mean ± SD, n = 9 from 3 independent experiments (3 technical replicates per experiment). Student’s two-sided t-test was used to determine significance. **G** Overview of WT (mCherry) and M41V in vitro competition experiment. Cells were mixed 50:50 and cultured with DMSO or TAK-243 (7.5 nM) for 6 days and chimerism was determined by the proportion of mCherry expressing WT cells. **H** Cell growth analysis of WT (mCherry) and M41V cells mixed at 50:50 ratio and cultured with DMSO or TAK-243 for 6 days. Mean ± SD, n = 3 independent experiments. Student’s two-sided t-test was used to determine significance. **I** Proportion of cells expressing Annexin V after DMSO or TAK-243 (7.5 nM) treatment for 72 hours. Mean ± SD, n = 3 independent experiments. Student’s two-sided t-test was used to determine significance. **J** Representative flow plot of Annexin V staining as quantified in (**I**). *P < 0.05; **P < 0.01; ***P < 0.001.

Since ubiquitin activation activity was perturbed in UBA1^M41V^ cells, we hypothesized that mutant cells would be sensitive to further loss of E1 enzyme activity. To test this, we used an shRNA approach to knock down UBA1 in the isogenic WT and M41V cells. Cells expressing either a control shRNA (shControl) or an shRNA targeting UBA1 (shUBA1) were isolated using fluorescence-activated cell sorting. The shRNA knockdown resulted in a > 90% reduction in UBA1 protein levels in both WT and M41V cells (Fig. 3A). Interestingly, this substantial reduction in UBA1 protein led to only a 15% decrease in total ubiquitination in WT cells, whereas total ubiquitination in M41V cells was not consistently reduced (Fig. 3A, Supplementary Fig. 3A), suggesting that chronic expression of UBA1 M41V mutations may result in compensatory mechanisms to maintain ubiquitination. To evaluate whether the loss of UBA1 affected cell growth, shControl- and shUBA1-expressing cells were cultured and counted over one week. As expected (see Fig. 1H), M41V cells expressing the control shRNA exhibited a moderate 25% growth reduction compared to WT cells (Fig. 3B). However, knockdown of UBA1 significantly impaired growth, causing a ~ 40% reduction in WT cells and a 20% reduction in M41V cells when normalized to their respective controls (Fig. 3B). While cells expressing shUBA1 were capable of growth in liquid culture, colony-forming potential in methylcellulose was completely ablated in both WT and M41V cells following UBA1 knockdown (Fig. 3C). These findings suggest that UBA1^M41V^ cells remain highly dependent on E1 ubiquitin function.

### UBA1^M41V^ cells are preferentially sensitive to the E1 inhibitor TAK-243

We demonstrated that UBA1 mutant cells were sensitive to loss of UBA1 expression (Fig. 3A–C). To extend these observations, we tested whether M41V cells were preferentially sensitive to the E1 ubiquitin activating enzyme inhibitor TAK-243 [58]. Consistent with prior studies [36, 37, 59], M41V cells (IC50 = 3.8 nM) exhibited reduced viability when treated with TAK-243 compared to isogenic WT cells (IC50 = 11.8 nM) (Fig. 3D). However, exposure to TAK-243 for 7 days in liquid culture did not result in a significant difference in cell growth between WT and M41V cells (Fig. 3E). We next assessed the effects of TAK-243 on the clonogenic potential of UBA1 mutant cells. Unlike growth in liquid culture, TAK-243 treatment (7.5 nM) had no impact on the clonogenic potential of WT cells but caused a significant ~40% reduction in colonies formation of M41V mutant cells treated with TAK-243 compared to treated WT cells (P = 0.002) (Fig. 3F). Increasing the TAK-243 concentration by less than 2-fold (10 nM) resulted in a strong reduction in clonogenic potential in both WT and M41V cells (Supplementary Fig. 3B), indicating a narrow therapeutic window for TAK-243 efficacy in this VEXAS model.

To further evaluate the sensitivity of mutant cells to TAK-243, we performed a cellular competition assay by mixing mCherry-expressing WT cells with mCherry-negative M41V cells at a 1:1 ratio and culturing them with either TAK-243 or vehicle control for 6 days (Fig. 3G). mCherry levels were monitored using flow cytometry to track the ratio of WT to M41V mutant cells (Fig. 3G). In the DMSO control, WT chimerism increased slightly but insignificantly, from 52% on day 0 to 57% on day 6. In contrast, TAK-243 treatment caused a significant increase in WT chimerism, rising from 52% on day 0 to 77% by day 6 (P = 0.02) (Fig. 3H). Given the strong reduction in relative cell growth of M41V cells compared to WT cells, we suspected that TAK-243 treatment preferentially induced apoptosis in M41V mutant cells. Cells treated with TAK-243 for 72 hours were stained with Annexin V to assess apoptosis. TAK-243-treated WT cells showed no significant increase in Annexin V expression, whereas TAK-243-treated M41V mutant cells exhibited a significant 4.8-fold increase in Annexin V+ cells compared to WT cells (P = 0.02) (Fig. 3I, J). These findings indicate that inhibition of E1 ubiquitin enzyme activity with TAK-243 results in preferential suppression of UBA1 mutant cells.

### TAK-243 preferentially inhibits UBA6 activity as compared to UBA1

**Fig. 4:**
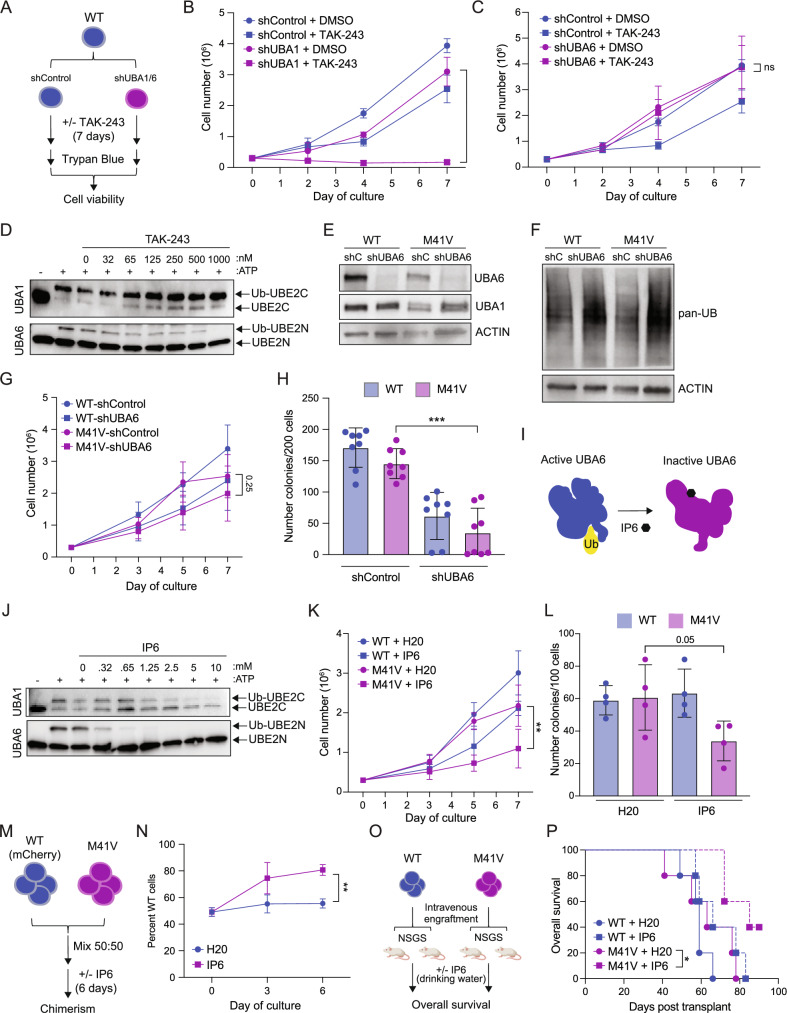
UBA1-mutant cells are sensitized to inhibition of UBA6. **A** Overview of growth curve analysis of THP1 cells expressing shRNA control (shControl, shC) or shRNAs targeting UBA1 (shUBA1) or UBA6 (shUBA6) and then cultured in DMSO or TAK-243 (7.5 nM) for 7 days. **B** Cell growth analysis of THP1 cells expressing shControl (shC) or shUBA1 and cultured in DMSO or TAK-243. Mean ± SD, n = 3 independent experiments. Student’s two-sided t-test was used to determine significance. **C** Cell growth analysis of THP1 cells expressing shControl (shC) or shUBA6 and cultured in DMSO or TAK-243. Mean ± SD, n = 3 independent experiments for shControl and n = 2 independent experiments for shUBA6. Student’s two-sided t-test was used to determine significance. **D** Immunoblot of a thioester assay of UBA1 and UBA6 activity with TAK-243 treatment. UBA1 was mixed with ATP, ubiquitin and UBE2C, an E2 conjugating enzyme. UBA6 was mixed with ATP, ubiquitin, and UBE2N, an E2 conjugating enzyme. UBA1 and UBA6 activity was determined by the ability to conjugate Ub onto the respective E2. **E** Immunoblot analysis of UBA6 and UBA1 in WT and M41V cells expressing shControl or shUBA6. **F** Immunoblot analysis of total ubiquitin (Ub) in WT and M41V cells expressing shControl or shUBA6. **G** Cell growth analysis of WT and M41V cells expressing shControl or shUBA6. Mean ± SD, n = 6 independent experiments. Student’s two-sided t-test was used to determine significance. **H** Quantification of clonogenic potential of WT and M41V cells expressing shControl or shUBA6. Mean ± SD, n = 8 from 4 independent experiments (2 technical replicates per experiment). Mann-Whitney test was used to determine significance. **I** Diagram of UBA6 showing active (closed) form and inactive (open) caused by allosteric inhibition of inositol hexaphosphate (IP6). **J** Immunoblot analysis of thioester of UBA1 and UBA6 activity with IP6 treatment. **K** Cell growth analysis of WT and M41V cells cultured in vehicle (H_2_O) or IP6 (5 mM). Mean ± SD, n = 6 independent experiments. Student’s two-sided t-test was used to determine significance. **L** Quantification of clonogenic potential of WT and M41V cells pre-treated for 7 days with vehicle (H_2_O) or IP6 (5 mM). Mean ± SD of 4 independent experiments. Student’s two-sided t-test was used to determine significance. **M** Overview of chimerism experiment in which a 50:50 mixture of WT and M41V cells were cultured in vehicle (H_2_O) or IP6 (5 mM) and mCherry proportions were determined to assess sensitivity to IP6 treatment. **N** Analysis of chimerism experiment described in (**M**). **O** Outline of mouse xenograft experiment. WT or M41V cells were engrafted into NSGS mice. 30 days post-transplant, mice were separated into two treatment arms of H_2_O or 1% Phytic acid (IP6) and overall survival was measured. **P** Kaplan-Meier survival curve of the experiment outlined in (**O**) (n = 5 mice per group). Log-rank Mantel-Cox was used to determine significance. *P < 0.05; **P < 0.01; ***P < 0.001.

Humans have two E1 ubiquitin-activating enzymes, UBA1 and UBA6, which catalyze the first step in the ubiquitination process by initiating the attachment of ubiquitin to E2 conjugating enzymes [3]. UBA1 serves as the dominant E1 enzyme for ubiquitin activation in human cells, whereas UBA6 plays a minor role in overall ubiquitination [31]. Since TAK-243 has demonstrated inhibitory activity against both UBA1 and UBA6 [58], we sought to determine whether the preferential sensitivity of M41V cells to TAK-243 was due to the inhibition of UBA1 or UBA6. To address this, we first tested whether shRNA knockdown of UBA1 or UBA6 would further sensitize WT THP1 cells to TAK-243 (Fig. 4A). As expected, either TAK-243 treatment or UBA1 knockdown alone caused a modest reduction in cell growth compared to vehicle or shRNA controls (Fig. 4B). Strikingly, combining TAK-243 treatment with UBA1 knockdown resulted in a complete ablation of cellular growth (Fig. 4B), suggesting that TAK-243 strongly inhibited UBA6 activity in these cells. Concordantly, TAK-243 treatment in UBA6 knockdown cells did not lead to further reductions in cell growth, further confirming that TAK-243 preferentially inhibited UBA6 (Fig. 4C). To further investigate the inhibitory activity of TAK-243 on E1-mediated ubiquitin conjugation, we performed a thioester assay to evaluate the functional ability of UBA1 and UBA6 to ubiquitinate an E2 conjugating enzyme. UBA1 or UBA6 were incubated with ATP, ubiquitin, and an E2 enzymes: UBE2C, a UBA1-dependent E2, or UBE2N, an E2 that functions with either E1 enzyme. Functional E1 activity was assessed by the formation of ubiquitin conjugates in the absence or presence of TAK-243. Consistent with our cell culture experiments, TAK-243 preferentially inhibited UBA6 conjugation activity compared to UBA1 (Fig. 4D). These findings indicate that TAK-243 more strongly inhibits UBA6 as compared to UBA1.

### UBA1^M41V^ cells are sensitized to genetic and pharmacological inhibition of UBA6

To investigate the effect of UBA6 inhibition, we first assessed the impact of shRNA-mediated UBA6 knockdown in the isogenic VEXAS cell lines. Similar to knockdown of UBA1 (Fig. 3A), shRNA -mediated knockdown of UBA6 resulted in a ~ 90% reduction in UBA6 protein levels (Fig. 4E). UBA6 knockdown led to increased UBA1 expression in WT cells but caused no change in UBA1 expression in M41V cells (Fig. 4E, Supplementary Fig. 4D). Interestingly, UBA6 knockdown caused a notable increase in total ubiquitination in both WT and M41V cells (Fig. 4F, Supplementary Fig. 4E), suggesting that there is functional compensation between UBA1 and UBA6 at least in human monocytic cells. Since loss of UBA1 and UBA6 did not significantly inhibit ubiquitination (Figs. 3A, 4F) we wanted to investigate the extent to which UBA1 and UBA6 may functionally overlap. We knocked down UBA1 or UBA6 in WT THP1 cells using shRNAs and performed RNA sequencing to identify differentially expressed genes compared to a scramble control. Surprisingly, while the number of upregulated and downregulated genes were similar between shUBA1 and shUBA6 cells, there was only 16% overlap in upregulated genes and 13% overlap in downregulated genes (Supplementary Tables 7, 8). This suggests that UBA1 and UBA6 have largely distinct functional roles in AML. To determine whether loss of UBA1 or UBA6 leads to similar pathway dysregulation, we analyzed the top 20 upregulated KEGG pathways. Notably, only 2 KEGG pathways were shared between UBA1 and UBA6 knockdown cells (Supplementary Fig. 4F). As this data suggested a unique functional role for UBA6, we next asked whether UBA6 loss is preferentially detrimental to UBA1 mutant cells. Knockdown of UBA6 similarly reduced cell growth in both WT and M41V cells compared to controls (Fig. 4G). However, UBA6 knockdown preferentially inhibited the clonogenic potential of M41V cells as compared to WT cells (75% +/- 13.7 vs 64% +/- 13.3) (Fig. 4H), indicating that UBA1 mutant cells have an acquired dependency on UBA6 function.

Since UBA6 inhibition may present as a novel targeted therapeutic strategy for VEXAS, we explored an orthogonal small-molecule approach to inhibit UBA6. Since selective clinical-stage UBA6 inhibitors have not been reported, we decided to evaluate inositol hexaphosphate (IP6), which has been confirmed to allosterically inhibit UBA6 by preventing its conformational activation [60] (Fig. 4I). We were also intrigued with IP6, commonly known as phytic acid, as it is abundant in grains and legumes and has been previously shown to exhibit anti-inflammatory activity [61, 62]. To confirm the specificity of IP6, we first evaluated its inhibition of UBA1 and UBA6 in biochemical assays. While 5 and 10 mM IP6 caused a partial reduction in UBA1 activity, IP6 completely ablated UBA6 activity at 2.5 mM in thioester assays, confirming its potency as a UBA6-specific inhibitor (Fig. 4J).

We next treated WT and M41V cells with IP6 and measured cell viability using CellTiter-Glo. Mutant M41V cells exhibited greater sensitivity to IP6 compared to WT cells (4.7 mM vs 5.9 mM IC50) (Supplementary Fig. 4A). Moreover, WT and M41V cells cultured in IP6 for one week showed a ~ 30% reduction in growth for WT cells and a ~ 50% reduction in growth for M41V cells (P = 0.004) (Fig. 4K). Similarly, IP6 treatment reduced colony formation by ~50% in M41V cells (P = 0.05), whereas WT cells maintained their clonogenic potential under the same conditions (Fig. 4L). Furthermore, the effects of IP6 was assessed in competition assays using WT and M41V cells, as in Fig. 3 (see panels G and H). Similar to TAK-243 treatment, we observed a significant increase in WT cell chimerism, increasing from 49% at day 0 to 81% at day 6, confirming that IP6 selectively targeted M41V cells (P = 0.0012) (Fig. 4M, N). Surprisingly, IP6 treatment did not increase Annexin V+ cells in either WT or M41V cells (Supplementary Fig. 4B, C). Given the strong reduction in cell growth caused by IP6, we next evaluated its effect on the cell cycle in WT and M41V cells. Isogenic cells were cultured with IP6 or vehicle control for 48 hours prior to cell cycle analysis. While IP6 treatment did not induce significant cell cycle changes in WT cells, M41V cells treated with IP6 showed a significant decrease in S-phase cells and an expansion of the sub-G1 population, a pre-apoptotic phase, which likely contributed to the increased sensitivity observed (Supplementary Fig. 4G, H). To further explore the potential therapeutic relevance of UBA6 inhibition for the treatment of VEXAS syndrome, we investigated the in vivo efficacy of IP6 in a xenograft model. Briefly, WT or M41V THP1 cells were transplanted into NSGS mice. Thirty days after engraftment, WT and M41V mice were randomized into two treatment groups: one receiving regular water and the other receiving water supplemented with 1% IP6. Overall survival was monitored across the four groups. There was no difference in median survival amongst the WT control and IP6 groups (59 and 66 days, respectively). In contrast, we observed a significant difference (p = 0.01) in median survival between M41V control and IP6 groups, with median survival of 63 and 85 days respectively. Collectively, these findings reveal that UBA1 mutations create an acquired dependency on UBA6, which can be exploited through UBA6 inhibition.

## Discussion

VEXAS syndrome is an acquired disorder characterized by systemic inflammation, multi-organ involvement, and high mortality. Mutations in the E1 ubiquitin enzyme, UBA1, are causative, yet the mechanisms by which mutated UBA1 drives disease pathogenesis remain unclear [23–28]. In this study, we describe a new human cell model of VEXAS syndrome, engineered by introducing the common M41V UBA1 mutation into the male monocytic cell line, THP1. This model replicates key features of the disease, including increased vacuolization and UBA1 isoform changes observed in patients. However, contrary to expectations, we do not observe a global reduction in ubiquitination. This aligns with observations in patient samples, where total ubiquitination is only mildly reduced ( ~ 20%) [23], suggesting that UBA1 mutations may alter specific subsets of ubiquitinated substrates rather than ubiquitination globally.

Systemic inflammation is a hallmark of VEXAS syndrome, yet the link between UBA1 mutations and inflammatory signaling remains poorly defined [26]. Our VEXAS cell model revealed altered ubiquitination of key inflammatory mediators but also a surprising transcriptomic downregulation of immune-related genes. This may reflect an adaptive mechanism in UBA1-mutant clones to survive chronic inflammatory stress. Supporting this, recent reports indicate that mutant UBA1 drives inflammatory stress and loss of wild-type HSPC clones [63]. Similar mechanisms of inflammation-mediated wild-type HSPC depletion have been demonstrated in models of clonal hematopoiesis of indeterminate potential [64] and MDS [19, 56]. We hypothesize that VEXAS pathogenesis involves UBA1-mutant HSPC dysregulation, where inflammation selectively depletes wild-type clones while mutant clones suppress inflammatory responses, enabling their survival under stress.

We further hypothesized that UBA1 mutations sensitize VEXAS cells to additional loss of E1 enzyme activity. To test this, we employed shRNAs and small-molecule inhibitors targeting both UBA1 and UBA6, the latter being the only other mammalian ubiquitin E1 activating enzyme. Surprisingly, UBA1 loss resulted in minimal changes to total ubiquitination, suggesting compensatory activity by UBA6. Conversely, UBA6 loss unexpectedly increased total ubiquitination, possibly due to compensatory upregulation of UBA1. These findings suggest functional overlap between the two enzymes, despite animal knockout studies indicating distinct roles for UBA1 and UBA6 [65, 66]. We observe limited overlap of differentially expressed genes in UBA1 and UBA6 deficient cells. It is plausible that while both enzymes compensate for ubiquitination linked to protein degradation, they possess unique functional ubiquitination profiles that cannot fully substitute for each other.

Our study identified UBA6 inhibition as a novel vulnerability in UBA1-mutant cells. Although TAK-243, an E1 inhibitor, preferentially suppresses UBA1-mutant cells, our data indicate that this effect is due to TAK-243-mediated inhibition of UBA6 rather than UBA1. This is consistent with biochemical assays showing that TAK-243 is a more potent inhibitor of UBA6 than UBA1 [58]. Currently, selective small-molecule inhibitors targeting UBA6 are not available. However, phytic acid, an allosteric inhibitor of UBA6 [60] with anti-inflammatory properties, suppresses UBA1-mutant cells in our model system. As phytic acid is abundant in grains and legumes [61, 62], it could potentially be evaluated in VEXAS patients. While UBA6 has not been directly implicated in hematological malignancies, our findings suggest that UBA6 compensation may be involved in other cancers with dysregulated ubiquitin. These findings highlight UBA6 as a unique therapeutic vulnerability in VEXAS, though further studies are needed to confirm this potential.

UBA6 is unique among E1 activating enzymes due to its dual substrate specificity for ubiquitin and the ubiquitin-like protein FAT10^67^. FAT10 modifications promote rapid proteasomal degradation, and under physiological conditions, UBA1 is thought to ubiquitinate >99% of proteins [31]. Thus, UBA6 is hypothesized to primarily function through FAT10-mediated pathways. Notably, FAT10 expression is normally restricted to immune cells but is induced during high inflammatory stress [67]. This raises the possibility that under excessive inflammation, common in many cancers [68], hematopoietic cells may utilize the UBA6/FAT10 axis instead of the UBA1/Ub axis. In VEXAS syndrome, dysregulation of UBA1 may drive dependency on the UBA6/FAT10 axis. Further studies are warranted to explore this dependency and its therapeutic implications.

In conclusion, our findings provide new insights into the molecular mechanisms of VEXAS syndrome. While UBA1 mutations do not globally reduce ubiquitination, they may selectively alter substrate-specific ubiquitination, contributing to disease pathogenesis. The functional interplay between UBA1 and UBA6 offer new avenues for therapeutic targeting in VEXAS.

## Materials and methods

### Cells

HEK293T cells were provided from S. Wells (CCHMC). THP1 were purchased from ATCC. All cells were authenticated by STR Profiling Service from ATCC. HEK293T cells were cultured in DMEM media (Fisher, Cat# SH30022FS) with 10% FBS and 1% penicillin/streptomycin. THP1 were cultured in RPMI-1640 media (Fisher, Cat# MT10040CV) with 10% of FBS and 1% penicillin/streptomycin.

### Reagents

TAK-243 purchased from Selleckchem (cat # S8341). Phytic acid (IP6) was purchased from EMD Millipore Sigma Aldrich (cat # P8810). All LC-MS grade solvents were obtained from J.T. Baker (Fisher Scientific).

### AAV vector and virus production

Gene editing vectors were derived from pAAV-MCS2 [a gift from Steve Jackson (Addgene plasmid #46954)]. The pAAV.M41VUBA1.LoxP.mCherry plasmid was generated with *UBA1* homology arms containing ~1.6 kb of homology comprising coordinates chrX: 47,198,198 – 47,199,787 (hg38). Each homology arm was ~800 bp in length and contained a LoxP sequence and was synthesized as a gBlock [69]. The right homology arm contains the M41V mutation at genomic coordinate 47,199,051(A > G, hg38) or the wild-type sequence for the M41M control vector. The PGK.mCherry.WPRE.SV40 cassette was generated by PCR amplification of a PLKO.1 TRC cloning vector with mCherry substituted for puromycin using primers FP: 5’ – GGGGTTGGGGTTGCGCCTTTT and RP: 5’ – TAAGATACATTGATGAGTTTGGACAAACCACA. pAAV-MCS2 was digested with NotI-HF and MluI-HF (NEB) and Gibson Assembly (NEB) was used to insert the two homology arms and the PGK.mCherry.WPRE.SV40 cassette. AAV was made as previously described [70]. Briefly, pDGM6 (a gift from David Russell, Addgene plasmid #110660) and pAAV target vectors were transfected into HEK293Ts with PEI Max (Polyethylenimine “Max” MW: 40,000 (Polysciences)). Cells were collected 48 hours later and lysed. Crude lysates were filtered with a 0.22um filter before being added to cells.

### Generation of CRISPR knock-in cells

THP1 cells were transfected with an sgRNA: 5’ GCCGTTCTTGGCCATTCCCT together with Cas9 2NLS Nuclease (Synthego) using Neon transfection system (Invitrogen). Filtered AAV lysate was added at 20% of culture volume immediately after Neon transfection. After incubating the cells for 7 days, mCherry+ cells were isolated with fluorescence-activated cell sorting and single cell sorted into 96 well plates. RNA was isolated from individual clones using the Quick-RNA MiniPrep kit (Zymo Research cat # R1055) according to manufacturer’s protocol. cDNA was generated utilizing the High-Capacity cDNA Reverse Transcription Kits (Thermo Fisher cat#: 4368814) according to manufacturer protocol. The cDNA was then diluted prior to PCR amplification of the UBA1 locus with primers 5’ - TTGACCCCACCAAGGATGATG and 5’ -TGTCCAAGAAACGTCGCG). Positive WT and M41V clones were confirmed with sanger sequencing. After confirmation of positive clones, cells were transfected with Cre recombinase mRNA (Trilink Biotechnologies L-7211-100) using the Neon transfection system to remove the mCherry cassette. Successful removal of the mCherry cassette was confirmed with flow cytometry.

### shRNA vector generation and lentiviral production

For knockdown studies, UBA1 and UBA6 shRNAs were cloned into the pLKO.1 TRC cloning vector (Addgene: #10878). The puromycin gene in pLKO.1 was replaced with GFP.

shRNA control (shC) (Sigma cat# SHC202):

5’- CCGGCAACAAGATGAAGAGCACCAACTCGAGTTGGTGCTCTTCATCTTGTTGTTTTT

UBA1 shRNA (shUBA1) TRCN0000004003:

5’ - CCGGCCTGGGATGTCACGAAGTTAACTCGAGTTAACTTCGTGACATCCCAGGTTTTTG

UBA6 shRNA (shUBA6) TRCN0000425327:

5’ – CCGGCCATTCCAATTGTAGTATTTACTCGAGTAAATACTACAATTGGAATGGTTTTTG

For production of lentiviral particles, lentiviral constructs were transfected together with packaging plasmids into 293 T producer cells using PEI Max MW: 40,000 (Polysciences). The media was exchanged with fresh media 24 hours later, and the supernatants were harvested 48 hours after the transduction. The harvested lentivirus containing supernatants were spinoculated with cells in addition of 0.8 ug/ml polybrene (Santa cruz cat#: sc-134220) at 32 C, 3000 rpm, 90 minutes.

### Analysis of cell vacuolization

30,000 cells from the THP1 VEXAS isogenic lines were cytospun onto 1% BSA coated slides (50ul of 1% BSA in PBS were spun unto slides for 2 minutes at 500 rpm) for 5 minutes at 350 rpm with low acceleration. Slides were dried overnight prior to staining with wright giemsa stain solution (Fisher cat#: 23264983) followed by diluted Giemsa Stain Buffer pH 6.8 (Fisher cat# 23-262236). Once dry, cells were coverslipped and images were acquired with a 60X oil objective on an upright, motorized Nikon NiE microscope with a 5×5 field view of the cells. These images were then quantified utilizing the ImageJ analysis software and vacuolated cells were manually counted. To minimize artifacts from cytospin collection, only larger vacuoles were counted in this analysis (Black arrows, Supplementary Fig. 1D).

### Colony forming assays

Colony-forming assay was done using Methocult H4434 Classic (Stem Cell Technologies, Cat# 04434) for human cells. 200-400 cells/ml of Methocult were plated in SmartDish 6 well plates (Stem Cell Technologies, Cat# 27371) and kept in 37 °C and 5% CO2. For TAK-243 experiments, DMSO or TAK-243 was added to methocult and vortexed immediately prior adding cells. Colonies were imaged and counted using a STEMvision (Stem cell technologies) automated cell counter after 14 days.

### Cell proliferation

For THP1 cells, cells were plated at 300 K/mL for all cell proliferation assays. For shRNA studies, cells were recovered for 2-4 days after FACS sorting prior to plating. For drug treated studies, cells were plated with DMSO or H_2_O vehicle controls or 7.5 nM or 10 nM of TAK-243 or 5 mM phytic acid. A full media and drug exchange was done on counting days and cultures were expanded to maintain cell densities under 1e6 cells/mL. Cells were counted on an automated CellDrop cell counter (DeNovix) using trypan blue exclusion assay over 7 days.

### Cell viability assays

For drug viability studies, serial dilutions of each compound or vehicle control were added in triplicate in a 96-well plate. 10,000 THP1 cells were added to each well and viability was measured with Promega’s CellTiter Glo 2.0 (cat# G7571) after 3-5 days according to manufacturer’s instructions. For Annexin V apoptosis analysis, cells were plated with the indicated drug amount and cultured for 1-3 days prior to staining. Cells were stained with 2ul of Annexin V eFluor® 450 (eBioscience cat# 48-8006-45) and 2ul of 7-AAD staining solution (eBiosciences cat# 00-6993-50) or 1ug/mL DAPI (Fisher cat# D1306). Acquisition was performed on a BD LSR/Fortessa.

### THP1 differentiation

To induce THP1 differentiation, cells were cultured with 80 ng/mL Phorbol 12-myristate 13-acetate (PMA; medchem express cat # HY-18739) or DMSO vehicle control for 3-5 days. Differentiation was measured by quantifying CD11b (1;100; eBioscience, cat# 25-0118-41) and CD14 (1:100; eBioscience, cat# 12-0149-41) expression via flow cytometry. Acquisition was performed on a BD LSR/Fortessa.

### Cell cycle analysis

For cell cycle analysis, cells were plated with TAK-243 or IP6 and the respective vehicle control (DMSO or H_2_O) for 48 hours. Cells were then incubated with 20 µmol/L EdU for 2 hours. Cells were then harvested and fixed with 4% PFA at room temperature and stained per the Click-iT EDU cell proliferation kit protocol (Thermo Fisher cat. #C10424) and 1ug/mL DAPI (Fisher cat# D1306). Acquisition was performed on a BD LSR/Fortessa.

### Immunoblot analysis

Protein extracts were prepared by lysing 1e6 cells in 50 uL sodium dodecyl sulfate (SDS) sample buffer containing benzonase (Millipore Sigma cat# 70746) and incubating on ice for 20 min. Samples were boiled at 95 °C for 5 min, separated by SDS-polyacrylamide gel electrophoresis (BIO-RAD), and transferred to nitrocellulose membranes (BIO-RAD cat# 162-0112). Immunoblot analysis was performed with the antibodies: UBA1 (Sigma, cat# HPA000289), UBA6 (Cell signaling, cat# 13386), pan-Ub (Abcam, cat# ab122), Pan-actin (Cell Signaling, cat# 4968), pRelA (Cell Signaling, cat# 8242), RelA (Cell Signaling, cat # 3033S), Vinculin (Cell Signaling, cat# 13901S) and His-tag (Cell Signaling, cat# 2365S). Blots were developed using ECL Western Blotting Substrate (Pierce, Cat #32106) or SuperSignal West Femto Substrate (Thermo Scientific, Cat #34096) and imaged on a BIO-RAD ChemiDoc Touch Imaging system.

### RNA sequencing

THP1 WT, M41V, scramble non-targeting control, UBA1 knockdown, and UBA6 knockdown cells were collected, and RNA was extracted using ZYMO RESEARCH Quick-RNA Miniprep kit (cat #R1055). After the confirmation of the RNA quality using Agilent 2100 Bioanalyzer, the libraries were prepared with polyA selection using the Truseq RNA Library Prep Kit, and the libraries were sequenced at an average depth of 30 M paired-end 100 bp nucleotide reads. After the quality of reads was examined using FastQC (v0.11.7, https://www.bioinformatics.babraham.ac.uk/projects/fastqc), paired-end reads were aligned against human (GRCh38) genome (iGenome,https://support.illumina.com/sequencing/sequencing_software/igenome.html) using HISAT2 (v2.0.5,http://daehwankimlab.github.io/hisat2). The raw gene counts were calculated using featureCounts (v1.5.2, http://subread.sourceforge.net/) and normalized using edgeR (v3.16.5, https://bioconductor.org/packages/release/bioc/html/edgeR.html) in iGEAK (https://pubmed.ncbi.nlm.nih.gov/30841853/). Differentially expressed genes were predicted using limma/voom (v3.30.6, https://bioconductor.org/packages/release/bioc/html/limma.html). Gene set enrichment analysis was performed as previously described [71]. For specific analyses, the ATF4 target gene list was obtained from Han et al. 2013 [54], the necroptosis gene list was obtained from Culver-Cochran et al. 2024 [12], and the NF-κB target gene list was adapted from https://www.bu.edu/nf-kb/gene-resources/target-genes/.

### Library preparation and DNA sequencing

Following initial sample dsDNA quality control for quantity with a Qubit fluorometer and size distribution with an Agilent Fragment Analyzer, 50 ng of gDNA is enzymatically fragmented using Twist Bioscience’s Library Preparation EF Kit 2.0 (Twist Bioscience, 104207). Index adapters (Twist Bioscience, 100577) are ligated onto the DNA fragments, and then amplified with 10 cycles of PCR. Libraries are purified with Binding and Purification beads (Twist Bioscience, 100983), quantified with a Qubit fluorometer, and checked again on a Fragment Analyzer. Up to 8 libraries of like organisms are pooled together with a total mass of 1500 ng/pool. Human DNA is then hybridized with Twist’s Comprehensive Exome probes (Twist Bioscience, 102032). Samples are hybridized for 30 minutes with Twist’s Fast Hybridization Reagents (Twist Bioscience, 104180) before being bound to streptavidin beads and washed with Twist’s Fast Hybridization and Wash Kit (Twist Bioscience, 104180). Following hybridization, hybridized pools are amplified with 8 cycles of PCR using primers and master mix from the Library Preparation Kit EF. Final libraries are purified with Twist’s Binding and Purification beads, quantified with a Qubit fluorometer, and their size checked with a Fragment Analyzer.

Libraries are sequenced on an Illumina NovaSeq X Plus, generating > 25 million 100 bp read pairs and analyzed using Illumina’s Dragen enrichment pipeline. Data Alignment and Analysis Methods Reads were aligned using a functionally equivalent pipeline, as previously defined [72]. Specifically, each read pair was separately aligned to the appropriate reference (GRCh38) genome using bwa-mem version 0.7.17 with the -M option. The resulting SAM files are first sorted and converted to BAM with Picard SortSam (V2.18.22), then all resulting BAMs are merged and duplicate marked with Picard MarkDuplicates (https://broadinstitute.github.io/picard/) using the “OPTICAL_DUPLICATE_PIXEL_DISTANCE = 2500” command line flag. Variants were filtered and subsetted using BCFtools (v1.9, https://samtools.github.io/bcftools/) and annotated with Variant Effect Predictor (VEP, https://useast.ensembl.org/info/docs/tools/vep/).

### Ubiquitin-enriched mass spectrometry

M41M WT or M41V mutant cells were prepared in triplicate and 20 million cells were collected and frozen at -70C. After probe sonication in a urea buffer as described in the Cell Signaling Technology (CST) protocol (cat #59322), a 660 nm protein assay was performed on the samples. 1.3 mg of protein was removed from each sample the volume was adjusted to 1 ml with the lysis buffer described in the protocol. The cells were lysed, reduced with DTT, alkylated with IAA and digested with trypsin, followed by ubiquitin enrichment using HS Ub/SUMO (K-e-GG) enrichment kit (Cell Signaling, cat #59322). After digestion with trypsin, the peptides were desalted and concentrated by running the samples through a C18 Sep-Pak (WAT051910) as described in the CST 5622S protocol and dried in a SpeedVac. Total ubiquitin (GG-K) peptides were enriched by passing the peptides over the HS (K-e-GG) magnetic beads according to the protocol. The eluted ubiquitin peptides were passed over a C18 stage tip for desalting and dried in a SpeedVac. The samples were reconstituted in 0.1% Formic acid (FA) and analyzed by nanoLC-MS/MS (Thermo Orbitrap Eclipse) with the LC, elution, and mass spectrometry parameters for label-free quantitation all documented previously [73]. The data were searched against a combined database of common contaminants and the uniprot *homo sapiens* database with Proteome Discoverer ver 3.0 using the Sequest HT search algorithm (Thermo Scientific) and a LFQ quantitation workflow incorporating the IMP-ptmRS node to calculate probabilities of site modifications. Ratios were calculated using the pairwise method with normalization to all ubiquitin peptides. P-values were calculated using the t-test background method of Proteome Discoverer. The final ubiquitin-peptide tables include only those peptides that were detected and quantified in both the WT and M41V samples. Significant differentially expressed proteins were analyzed using Enrichr [74–76] (https://maayanlab.cloud/Enrichr/) to identify enriched pathways.

### Total proteomics mass spectrometry

M41M WT or M41V MUT cells were cultured and three replicates of 2 million cells were collected from each cell line. The samples were solubilized in 100 μl of Thermo Easypep lysis buffer (A45735) with 1 μl of universal nuclease added. The samples were then reduced, alkylated and digested with LysC/trypsin according to the Easypep MS sample prep kit (A40006) instructions. The peptides were desalted using the columns provided in the Easypep MS sample prep kit according to the provided instructions and then dried in a speed vac. The samples were resuspended in 0.1% Formic acid (FA) and 10% of each sample was analyzed by nanoLC-MS/MS (Orbitrap Eclipse) using Label Free Quantitation (LFQ) parameter as documented previously [73]. The complete instrument parameters are provided as supplementary information. The results were searched against a combined contaminant database plus the swissprot homo sapiens database using the Sequest HT search algorithm and the LFQ quantitation workflow in Proteome discoverer ver 3.0 (Thermo scientific). Abundances were normalized to total peptides and ratios were calculated using the pairwise method. P values were calculated using the background-based method in PD. The final protein tables include only those proteins that were detected and quantified in both the WT and M41V samples. Significant differentially expressed proteins were analyzed using Enrichr [74–76] (https://maayanlab.cloud/Enrichr/) to identify enriched pathways.

### Thioester assay

For UBA1 (synthesized by Genscript) reactions, a master mix was created with 0.5uM His-UBE2C (UPBio, Cat#C1300), 10uM ubiquitin (UBPBio, cat# E1100), UPBio Buffer, 2 mM ATP (Sigma, cat# GE27-2056-01), glycerol (Fisher, cat# bp2291), and MillQ Water. For UBA6 (Abcam, cat# ab269092) reactions, a master mix was created with 0.5uM His-UBE2N (synthesized by Genscript), 50uM ubiquitin (UBPBio, cat# E1100), UPBio Buffer, 4 mM ATP (Sigma, cat# GE27-2056-01), glycerol (Fisher, cat# bp2291), and MillQ Water. If applicable drug dilutions and vehicle controls (DMSO or H_2_O) were added to the reactions (final volume 20uL). The reactions were incubated at 37 °C. The UBA1 reactions were incubated for 30 minutes, and the UBA6 reactions were incubated for 3 hours. Reactions were quenched with 20 uL of 2x Laemmli buffer with no additives (BioRad, Cat# 161-0737) and boiled for 5 minutes. Reactions were either frozen at -20 °C or immediately analyzed by immunoblot with a His-tag antibody (Cell Signaling, Cat# 2365S).

### Xenograft assays

Animals were bred and housed in the Association for Assessment and Accreditation of Laboratory Animal Care-accredited animal facility of Cincinnati Children’s Hospital Medical Center. The experiments in this study were performed in compliance with the Institutional Animal Care and Use Committee (IACUC) protocols (2022-0054). 300,000 WT or M41V THP1 cells were transplanted into unconditioned NSGS mice. After 30 days, mice were split into two treatment arms: H2O and NaOH buffered 1% phytic acid water. To make NaOH buffered 1% phytic acid, 20 mL of 5 N NaOH and 15 mL of 50% (v/v) of phytic acid (Millipore, Cat# 593648) were added to 720 mL of MilliQ water and autoclaved. The mice were monitored over time, and mice were sacrificed when they reached IACUC defined endpoints.

### Statistics

The number of cells and experimental replicates can be found in the figure legends. No power calculations were performed to determine sample size in this study. For mouse xenograft studies, mice animals received from the mouse core were randomly assigned to receive either WT or M41V cells. Researchers were not blinded to the group or treatment arms during the experiment. Comparison of two groups was performed using the Student’s *t* test (unpaired, two tailed) when sample size and data normality allowed. A P value less than 0.05 was considered significant. Unless otherwise specified, results are depicted as the mean ± standard deviation or standard error of the mean. All graphs and analysis were generated using GraphPad Prism software or using the package ggplot2 from R [77].

## Supplementary information

Supplemental Figures

Supplemental Tables

## Data Availability

The RNA sequencing data from this study are available on GEO (GSE294655). The WES data from this study are available on GEO (GSE294656). The proteomics data from this study are available on PRIDE [78] (Project accession: PXD063067). The ubiquitin-enriched proteomics data from this study are available on PRIDE (Project accession: PXD063068). Cell lines used in these studies are publicly available through commercial sources or may be made available from the authors upon written request and material transfer agreement approval. The authors are also glad to share guidance regarding protocols and assays used in these studies upon written request.
