# Supplementary material for: Characterization of E1 enzyme dependencies in mutant-UBA1 human cells reveals UBA6 as a novel therapeutic target in VEXAS syndrome
Source: Leukemia. 2025 Jun 30;39(8):1997–2009. doi: 10.1038/s41375-025-02671-x (PMC12310546; doi:10.1038/s41375-025-02671-x)
Supplement: Supplementary file 1 — Supplemental Figures [file 41375_2025_2671_MOESM1_ESM.pdf]

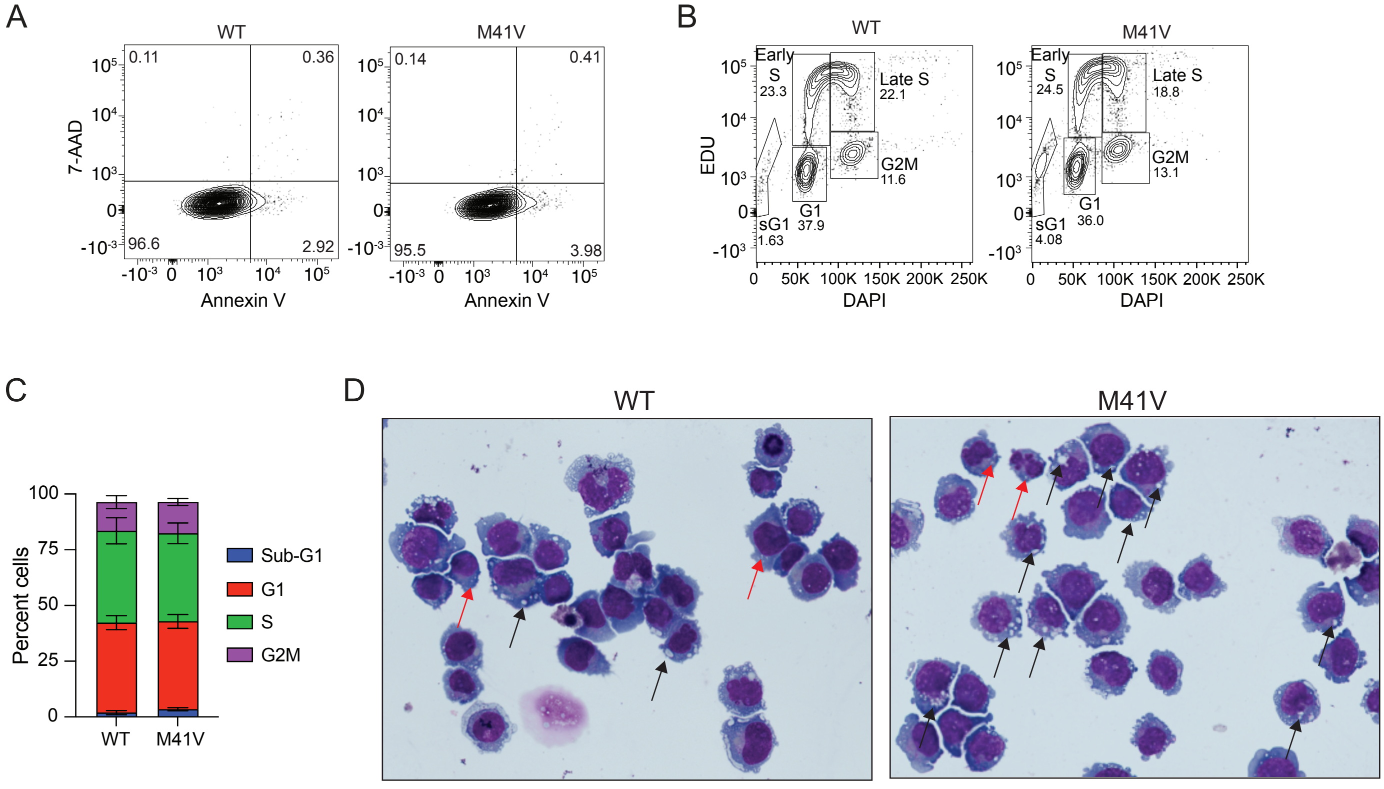

Supplemental Figure 1. Characterization of WT and M41V isogenic THP1 cells. (A) Representative flow plots of Annexin V staining of baseline WT and M41V cells. **(B)** Representative flow plots of cell cycle analysis of WT and M41V cells. **(C)** Quantification of cell cycle analysis of WT and M41V cells (from Panel B). Mean +/- SD of 3 independent experiments. **(D)** Representative cytospin images from WT and M41V cells. Black arrows indicate representative large vacuoles that marked cells labeled as vacuolated. Red arrows indicate cells with small vacuoles that were considered artifacts of cellular collection and cytospins and were ignored during vacuole quantification.

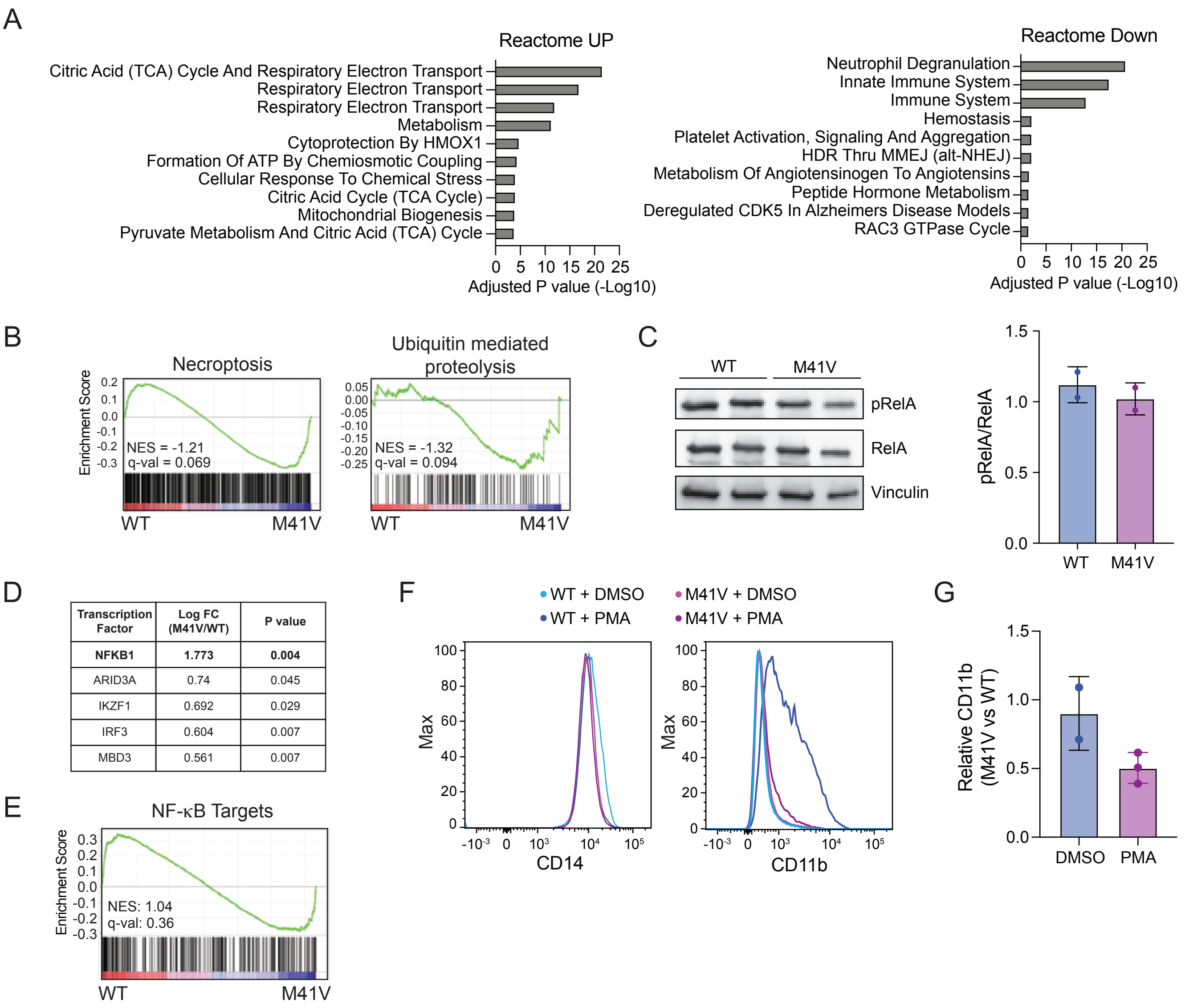

Supplemental Figure 2. Gene expression analysis and differentiation potential of WT and M41V THP1 cells. (A) Reactome pathway analysis of significantly upregulated and downregulated proteins from Fig 2B. **(B)** GSEA analysis of necroptosis related genes (Culver-Cochran et al, Nature Communications, 2024) or the KEGG ubiquitin mediated proteolysis geneset of the WT and M41V RNA sequencing data described in Fig 2H. **(C)** Immunoblotting of WT and M41V cells (two independent collections) for total and phosphorylated RelA/p65. Shown on the right is the quantification of baseline pRelA and RelA expression in WT and M41V cells. **(D)** Transcription factors that were signifcantly differentially expressed in M41V cells compared to WT cells in the proteomics data described in Figure 2B. **(E)** GSEA analysis of NF-κB targets (adapted from https://www.bu.edu/nf-kb/gene-resources/target-genes/) of WT and M41V RNA sequencing data in Figure 2H. **(F)** Representative CD14 and CD11b histogram plots of PMA-induced differentiation of WT and M41V THP1 cells after 96 hours. **(G)** Quantification of relative M41V CD11b expression compared to WT cells treated with DMSO or PMA for 96 hours. Mean +/- SD of n = 2 independent experiments for DMSO and n = 3 for PMA treated cells.

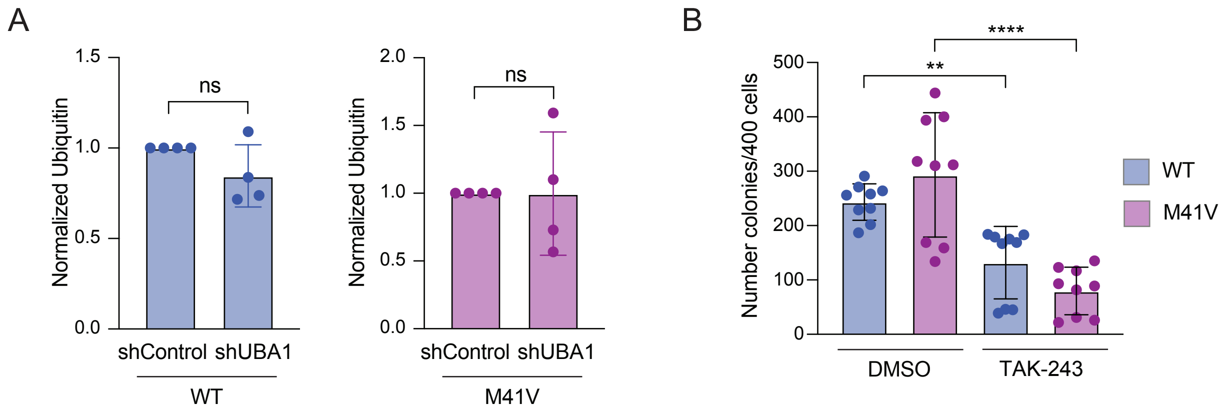

Supplemental Figure 3. Ubiquitination and colony forming potential of WT and M41V THP1 cells following inhibition of UBA1. (A) Quantification of normalized total ubiquitin in WT and M41V cells infected with shControl or shUBA1 vectors. Mean +/- SD of 4 independent experiments. Student's t-test was used to determine significance. **(B)** Quantification of clonogenic potential of WT and M41V cells treated with vehicle control (DMSO) or TAK-243 (10 nM) for 48 hours prior to plating into methylcellulose containing DMSO or TAK-243 (10 nM). Mean +/- SD, n = 9 from 3 independent experiments (3 technical replicates per experiment). Student's t-test was used to determine significance.

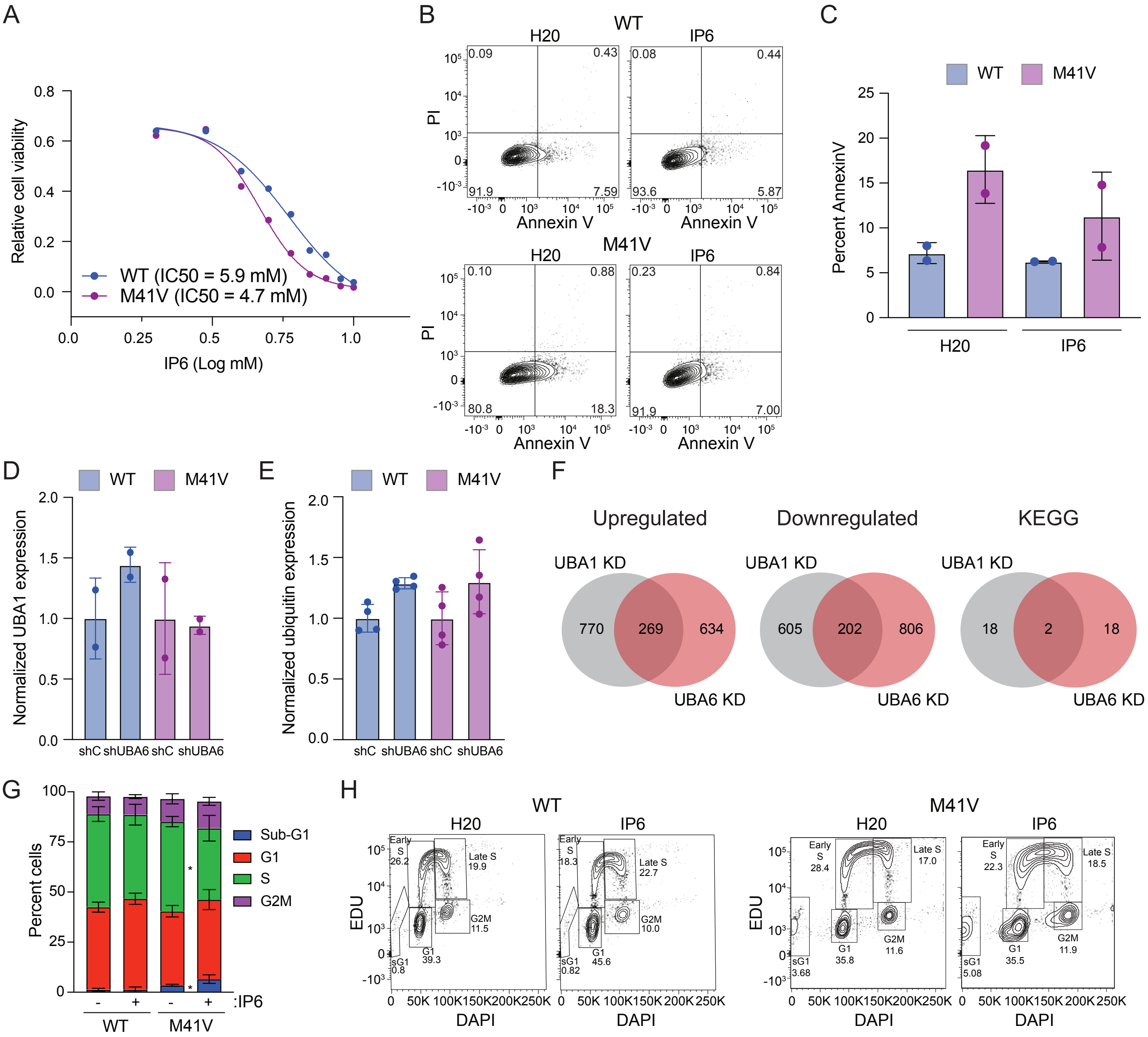

Supplemental Figure 4. UBA6 inhibition in WT and M41V THP1 cells. (A) Cell viability analysis using CellTiter-Glo of WT and M41V cells treated with IP6 for 5 days. Mean, n = 3 independent experiments. **(B)** Representative flow plots of Annexin V staining of WT and M41V cells treated with vehicle control (H2O) or IP6 (5 mM) for 72 hours. **(C)** Quantification of Annexin V staining of WT and M41V cells treated with vehicle control (H2O) or IP6 (5 mM) for 72 hours. Mean +/- SD, n = 2. **(D)** Quantification of immunoblot analysis of normalized UBA1 immunoblot expression in WT and M41V cells infected with shC or shUBA6. Mean +/- SD, n = 2 independent experiments. **(E)** Quantification of immunoblot analysis of normalized ubiquitin expression in WT and M41V cells infected with shC or shUBA6. Mean +/- SD, n = 4 independent experiments. **(F)** Venn diagram of significantly upregulated and downregulated genes and upregulated KEGG pathways in UBA1 and UBA6 shRNA knockdown cells. **(G)** Quantification of cell cycle analysis of WT and M41V cells treated with vehicle control (H2O) or IP6 (5 mM) for 48 hours. Mean +/- SD, n = 3 independent experiments. Student's t-test was used to determine significance. **(H)** Representative cell cycle flow plots of WT and M41V cells treated with vehicle control (H2O) or IP6 (5 mM) for 48 hours. *, $P < 0.05$; **, $P < 0.01$; ***, $P < 0.001$.
